# Bioactivities of Halometabolites from Marine Actinobacteria

**DOI:** 10.3390/biom9060225

**Published:** 2019-06-11

**Authors:** Noer Kasanah, Triyanto Triyanto

**Affiliations:** 1 Integrated Laboratory, Faculty of Agriculture, Universitas Gadjah Mada, Yogyakarta 55281, Indonesia; triyantoiwak@ugm.ac.id; 2 Department of Fisheries, Faculty of Agriculture, Universitas Gadjah Mada, Yogyakarta 55281, Indonesia

**Keywords:** marine actinobacteria, chemodiversity, antibacteria, anticancer, halometabolites

## Abstract

Natural halogenated compounds (halometabolites) are produced mainly by marine organisms, including marine Actinobacteria. Many commercially important compounds for pharmaceuticals contain halogen, and the halogen is responsible for the physical and chemical properties as well as bioactivities and toxicities. In the exploration of marine environment that is supported by advanced structure elucidation, varied panel bioassays and high-throughput screening have accelerated number of halometabolites isolated from marine Actinobacteria to date. The metabolites exhibited unique structures and promising bioactivities. This review focuses on the chemodiversity and bioactivities of marine halometabolites from marine Actinobacteria reported in the last 15 years (2003–2018).

## 1. Introduction

Halometabolites are a group of compounds contain halogen substituents (F, Cl, Br, I). To date, there are more than 5000 halogenated compounds with high degree of structural variability containing a single or several halogen atoms [1]. Halogen elements are found in several forms in nature. Chloride, iodine, and bromide salts are present in the oceans, while the Earth’s crust is rich in fluorine. Natural organohalogens (or halometabolites) are produced from two sources: abiogenic and biogenic. Natural abiogenic organohalogen is formed during geothermal processes such as volcano, hot springs, or earthquake. Biomass burning and soil chemistry have also contributed to the enormous number of abiogenic halometabolites [2]. Biogenic halometabolites are produced by bacteria, fungi, plants, marine invertebrates, and macroalgae [1,2,3,4,5,6,7,8]. 

Halometabolites in nature have several functions in physiological, biochemical, or defensive role for their host including communication (quorum sensing) and production of growth hormones, sex pheromone, toxin, or antibiotics. The role of substituent halogen in organic compounds is related to the bioactivity, bioavailability, and stability of the compounds. 

Chlorinated antibiotics were discovered from the exploration of soil Actinobacteria since the discovery of streptomycin from *Streptomyces griseus*. A number of drugs derived from Actinobacteria such as antibiotic and anticancer are on the market today. Chlorinated antibiotics such as chloramphenicol and vancomycin played important roles for the eradication of infectious diseases in human. Chloramphenicol is a broad-spectrum antibiotic used to treat bacterial infections. Chloramphenicol antibiotic is on the WHO (World Health Organization) list of essential medicine. Chlortetracycline is a member of the tetracycline family and produced by *Streptomyces aureofaciens.* Chlortetracycline was used clinically in 1948 and is used to prevent, control, and treat animal health problems and increase growth rate in chickens, turkeys, ducks, swine, calves, beef cattle, and others. Calicheamicin is a group of enediyne metabolite with iodine and has remarkable activity as anticancer produced by *Micromonospora echinospora.* Linking calicheamicin to monoclonal antibody is used for therapy of acute myeloid leukemia. Rebeccamycin is produced by *Streptomyces* sp. and is a derivate of staurosporine with chlor attached to it. Rebeccamycin showed in vitro antitumor activity at Inhibitory Concentration (IC_50_) 480 nM against P388 leukemia cells. Complestatin is a cyclic halogenated peptide produced by *Streptomyces lavendulae* anti and exhibited activity as HIV-1 integrase inhibitor. Vancomycin is a halogenated glycopeptide active against *Staphylococcus aureus* (including methicillin-resistant strains)*, S. epidermidis* (including multiple-resistant strains)*, Streptococcus pneumoniae* (including multiple-resistant strains)*, S. pyogenes, S. agalactiae, S. bovis, S. mutans, Clostridium spp., Listeria monocytogenes, Actinomyces spp., and Lactobacillus spp.* [9,10,11]. 

## 2. Research Methodology

In the studies on bioactive marine natural products, we look for the potential of marine Actinobacteria as halometabolites producers. This comprehensive review illustrates the chemistry and biological activities of halometabolites produced by marine Actinobacteria reported in the last 15 years (2003–2018). Mining and searching for data of compounds and bioactivities were obtained from reports in the database MarinLit, Google Scholar, ScienceDirect, Dictionary of Marine Natural Products, and Marine Natural Product Review. Herein, we grouped the halometabolites based on class of compounds. 

## 3. Halometabolites Isolated from Marine Actinobacteria

The marine environment is home for wide diversity of organisms and sources of structurally diverse secondary metabolites and drug leads. Halometabolites were produced mainly by marine organisms because seawater contained ion chloride and bromine. Marine organisms have the capability to oxidize bromide more easily than chlorine in the biosynthesis of organic compounds, thus bromometabolites are higher than chlorometabolites as observed in sponge and red algae [4,5,7].

**Figure 1:**
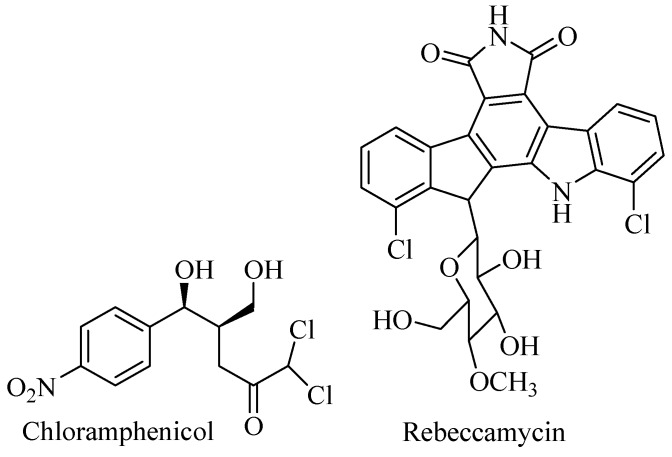

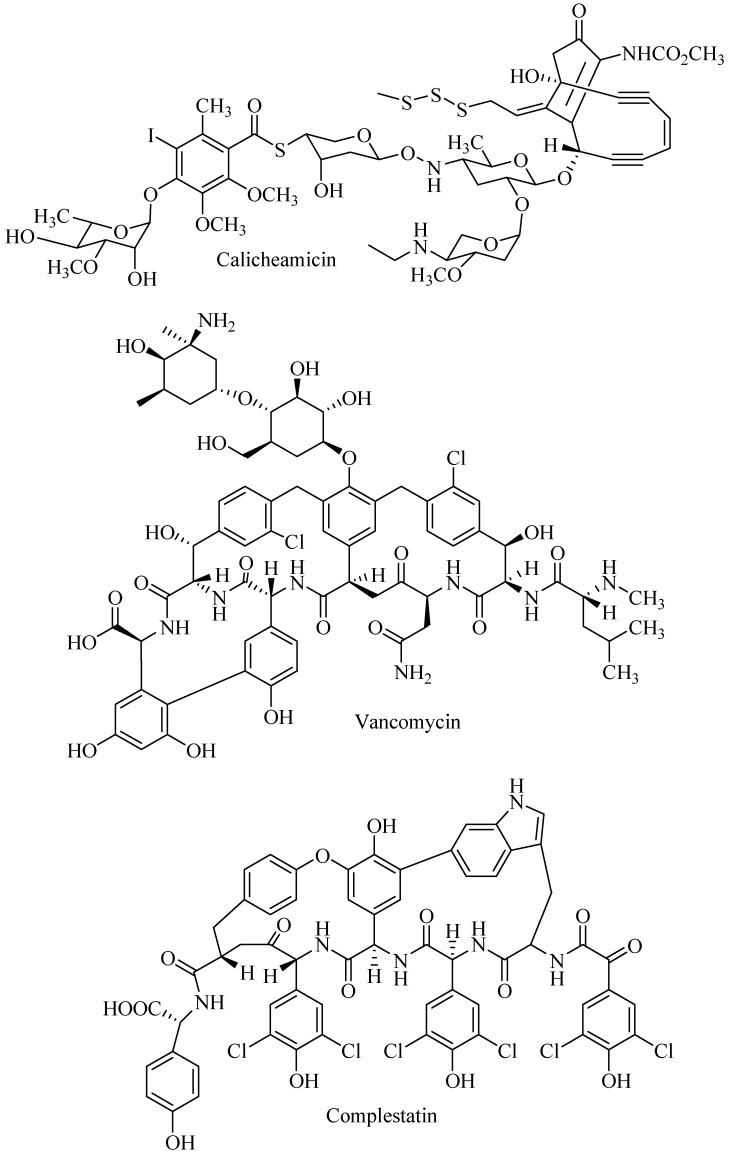
Halometabolites from terrestrial Actinobacteria.

The phylum Actinobacteria is Gram-positive bacteria with high G-C content in DNA. Terrestrial Actinobacteria has been explored for decades as sources of pharmacologically active compounds, and more than 70% of antibiotics used today are derived from Actinobacteria. Other bioactive compounds such as anticancer, antifungal, anthelminthic, antidiabetic, etc., were discovered from terrestrial Actinobacteria as well. Figure 1 shows diverse commercial halometabolites isolated from terrestrial Actinobacteria to prove Actinobacteria play important roles for biomedicine and biotechnology applications [9,12]. 

Marine environment is different from terrestrial so that marine Actinobacteria have special characteristic and adapted to stress in marine environment. As a result, marine Actinobacteria produce new type of secondary metabolites that differs from terrestrial one. Marine Actinobacteria can be found in any part of the ocean such as water column, sediment, deep sea, and in association with seaweed, sponges, and marine organisms [13,14,15]. Marine Actinobacteria have been explored and yielded structurally unique secondary metabolites with varied biological activities [15,16,17]. Searching for bioactive halometabolites was focused on many members of this genera such as *Streptomycetes, Actinoplanes, Nocardia*, and other rare Actinobacteria [18,19,20]. One study showed that marine Actinomycetes was a major producer of antibacterial compounds compared to Bacilli and Gammaproteobacteria [21]. 

### 3.1. Alkaloid

#### 3.1.1. Lynamicins

**Figure 2:**
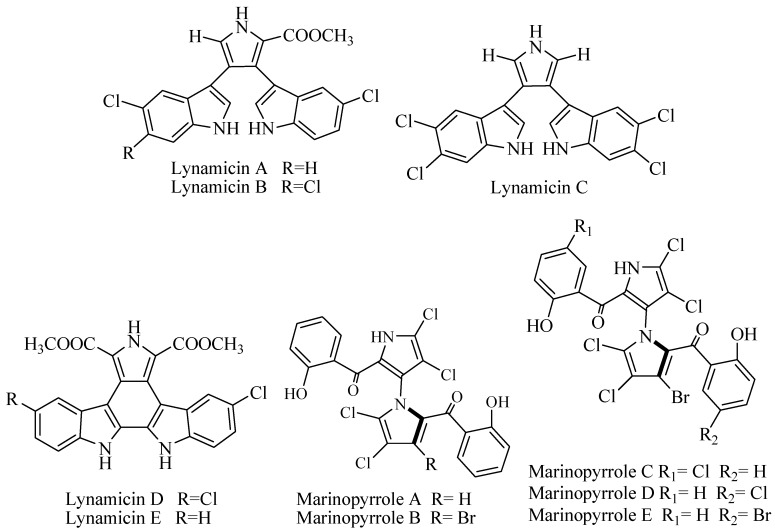

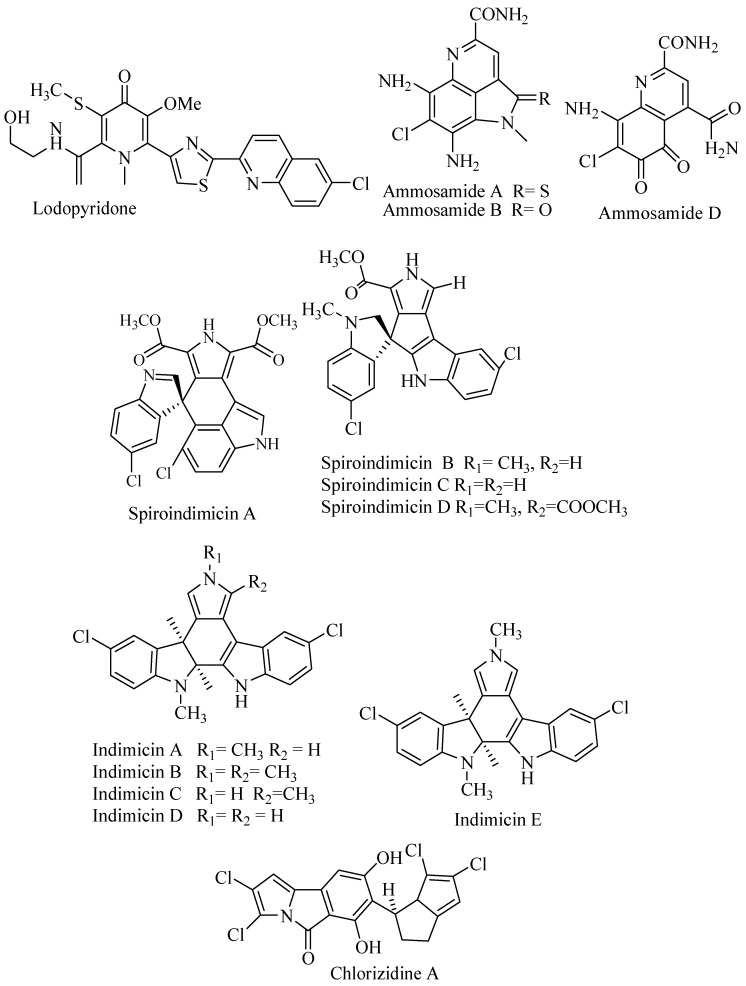
Halogenated alkaloid.

Marine Actinomycete NPS 12745 (*Marinispora* sp.) was isolated from marine sediment of Mission Bay, San Diego, coast yielded bisindole pyrrole compounds named lynamicins A–E (Figure 2). The series of compounds were tested against series of panel test bacteria that were resistant and sensitive to antibiotics. However, lynamicin E exhibited broad-spectrum activity and potency for treatment of nosocomial infection at Minimum Inhibition Concentration (MIC) 1.8–36 μg/mL [22].

#### 3.1.2. Marinopyrroles

An obligate marine Actinomycetes CNQ-418 related to *Streptomyces* was isolated from 51 m sediment of La Jolla, California, produced two unique halogenated metabolites with uncommon 1,3′-bipyrrole pharmacophore called marinopyrrole A and B (Figure 2). The compounds were active against methicillin-resistant *Staphylococcus aureus* at MIC_90_ 0.61 and 1.1 μM for marinopyrrole A and B, respectively. The IC_50_ against HCT-16 (human colon cancer cell line) for marinopyrrole A was 8.8 μM and marinopyrrole B was 9.0 μM [23]. Further examination of broth fermentation of *Streptomyces* strain CNQ-418 yielded marinopyrroles A–F. The compounds showed variation of substitution chlorine and bromine. Marinopyrrole A–C had significant activity against MRSA (methicillin-resistant *Staphylococcus aureus*) with MIC_90_ at less than 1 μg/mL [24]. Marinopyrrole A showed activity against *S. aureus* strains with MIC 0.188–1.5 μg/mL. This activity was better than available antibiotics vancomycin and linezolid. In addition, marinopyrrole A was active against *H. influenzae* at MIC 2 μg/mL. The toxicity against mammalian cell line was more than 20 times of the MIC value [25]. Marinopyrrole A is reported to be an antagonist of Myleoid Leukemia (Mcl-1), a member of the anti-apoptotic B-cell Lymphoma-2 (Bcl-2) family, which is a well-validated drug target for cancer treatment. The cell-based assay shows a high selectivity of marinopyrrole A. Treatment with marinopyrrole A inhibits the viability of K562 cells transfected with *Mcl-1* gene with Effective concentration (EC_50_) value of 1.6 μM. The selectivity is more than 40-fold greater over the cells transfected with *Bcl-XL* gene. Moreover, marinopyrrole A can decrease Mcl-1 expression by increasing the cleavage of caspase-3 and Poly (ADP-ribose) polymerase (PARP). Marinopyrrole A is also reported to completely restore the sensitivity of multidrug-resistant leukemia cells to ABT-737 [26]. 

#### 3.1.3. Lodopyridone

Lodopyridone (Figure 2) is a unique alkaloid isolated from an obligate marine *Saccharomonospora* CNQ-490 collected from sediment in La Jolla Submarine Canyon, California. The compound has interesting carbon skeleton properties with ethanolamine, thiomethyl with substitution of pyridine, thiazole, and chloroquinoline. The bioactivity was modest against cancer cell line HCT-116 at IC_50_ 3.6 μM, but there was no activity against MRSA [27]. 

#### 3.1.4. Ammosamide

Heteroaromatic alkaloids contain pyrroloquinoline ammosamide A and B (Figure 2) were isolated from *Streptomyces* CNR-698 from Bahama Island at 1618 m. Bioassay-guided fractionation yielded ammosamide A and B that showed cytotoxicity against HCT-116 at IC_50_ 320 nM [28]. Ammosamide D was isolated from marine *Streptomyces variabilis* SNA-020 from Sweetings Cay, Bahamas. The cytotoxicity of ammosamide D to human cell line HCT-116 was at IC_50_ 3.2–4.9 µM [29]. 

#### 3.1.5. Spiroindimicins

PCR-based screening of tryptophan dimerization gene has enabled to select the deep-sea *Streptomyces* SCS10 03032 from South China Sea produced bisindole alkaloid with unique spiro ring spiroindimicin A–D (Figure 2) along with lynamicin A and D. The compounds were evaluated for cytotoxic activity against cancer cell lines MCF-7, HepG2, B16, H460, and CCRF-CEM. Spiroindimicin A showed no inhibitory activity, while spiroindimicin B showed cytotoxicity against CCRF-CEM (IC_50_ 4 μg/mL), B16 (IC_50_ 5 μg/mL), and H460 (IC_50_ 12 μg/mL). Spiroindimicin C exhibited inhibition against HepG2 and H460 at IC_50_ 6 and 15 μg/mL respectively, while spiroindimicin B and D with property [5,5]spiro-ring showed moderate activity against HepG2, B16, and H460 [30]. 

#### 3.1.6. Indimicin

Deep sea *Streptomyces* sp. SCS10 03032 from South China produced chlorinated bisindole alkaloid indimicin A–E (Figure 2) along with lynamicin F and G. The antibacterial activity was tested against *E. coli* ATCC 25922, *S. aureus* ATCC 29213, *B. thuringiensis* SCSIO BT01, *B. subtilis* SCSIO BS01, and *C. albicans* ATCC10231 with MIC > 128 μg/mL and was considered as inactive. The cytotoxicity was tested against cancer cell line, and only indimicin B was active against MCF-7 cell line at IC_50_ 10 μM [31]. 

#### 3.1.7. Chlorizidine

An obligate marine *Streptomyces* strain CNH-287 was isolated from marine sediment San Clemente, California, produced chlorizidine (Figure 2). The compound revealed feature structure on 5H-pyrrolo[2,1-a]isoindol-5-one ring system that never been reported before. The cytotoxicity against HCT-116 was reported at IC_50_ 3.2–4.9 μM [32].

### 3.2. Terpene and Meroterpene

#### 3.2.1. Azamerone

**Figure 3:**
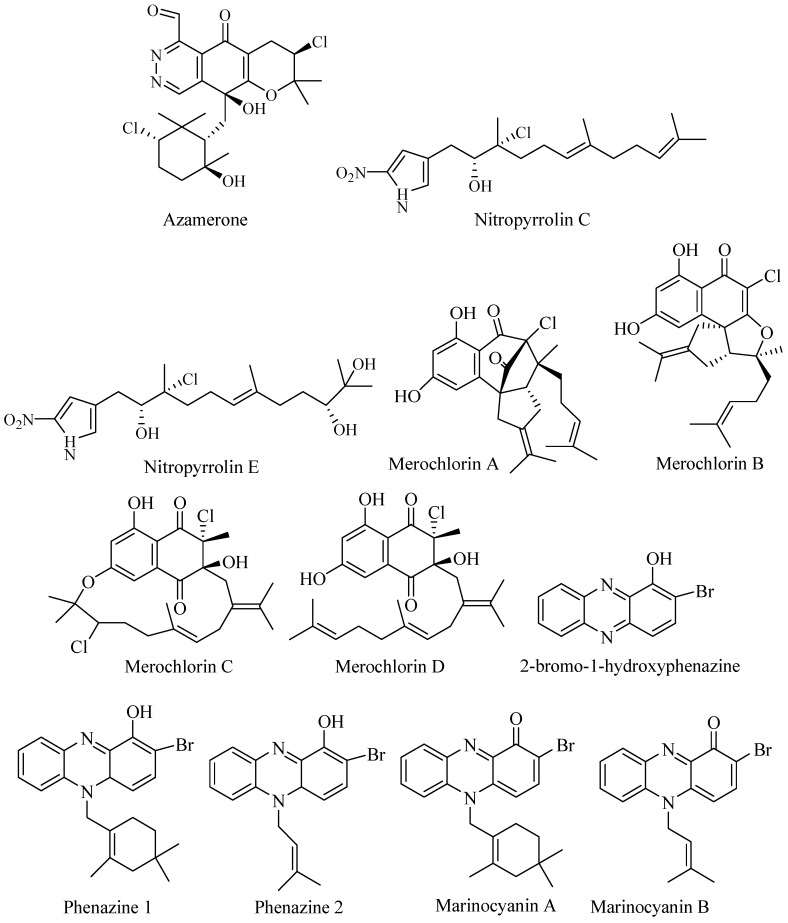

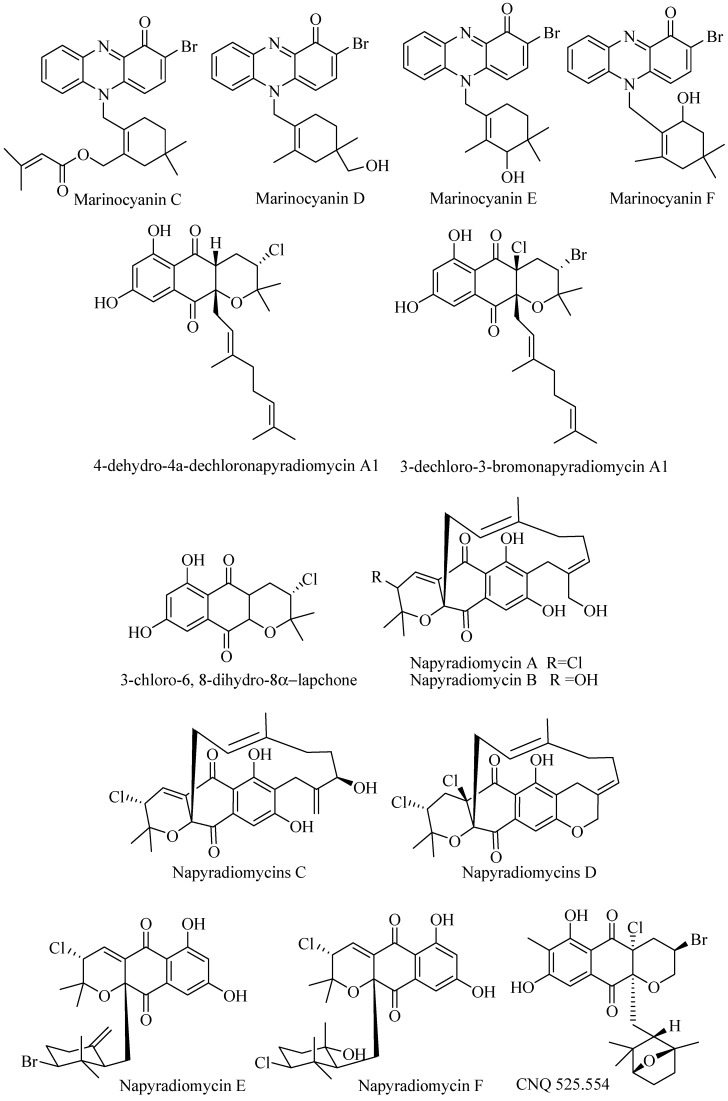

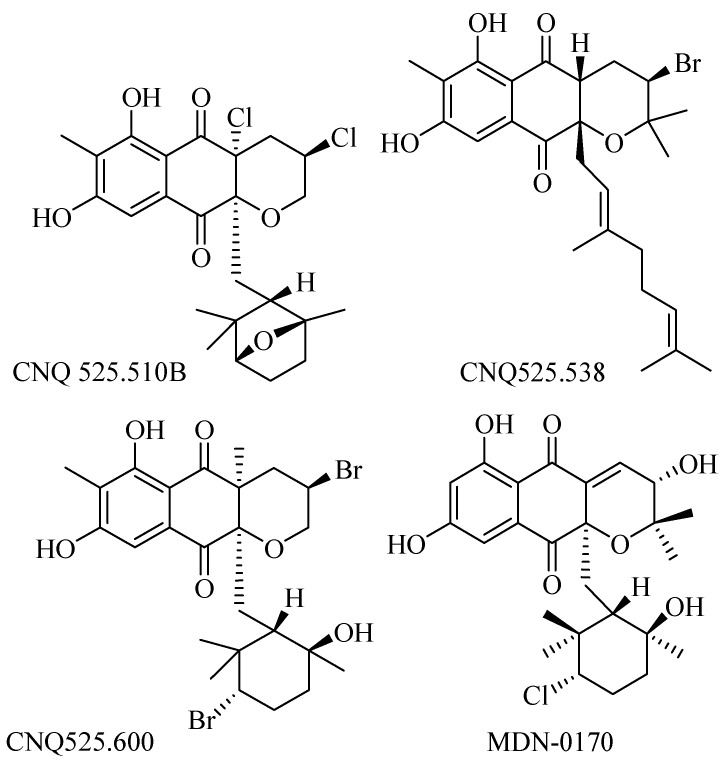
Halogenated terpene and monoterpene.

Azamerone (Figure 3) was isolated from saline culture of two strain *Streptomyces* MAR-4 and CNQ-766. The compound is meroterpenoid with phthalazinone ring with a side chain of 3-chloro-6-hydroxy-2,2,6-trimethyl-cyclohexyl-methyl (Figure 3). In vitro cytotoxicity assay against mouse splenocyte population of T-cells and macrophage showed weak potency at IC_50_ 40 μM [33].

#### 3.2.2. Nitropyrrolins

Marine Actinomycetes strain CNQ-509 isolated from marine sediment of La Jolla, California, produced a new set of nitrophyrrolins (Figure 3). The compounds were hybrid isoprenoid composed of linear sesquiterpenoid and α farnesyl nitropyrrole. Two compounds had chlorin moieties Nitropyrolins C and E. The compounds showed no activity against MRSA and cancer cell line HCT-116 [34].

#### 3.2.3. Merochlorins

Bioassay-guided fractionation has led to discover novel meroterpen merochlorins (Figure 3) from marine *Streptomyces* CNH-189 from coastal sediment in California. The compounds displayed unrelated skeleton to available antibacterial agents [35]. Merochlorin A was active against Gram-positive bacteria but inactive against Gram-negative bacteria and showed no cross-resistance to Gram-positive bacteria. Merochlorins A was active against MRSA, MSSA (methicillin-sensitive *Staphylococcus aureus*), VSSA (vancomycin-sensitive *Staphylococcus aureus*), and VRSA (vancomycin- resistance *Staphylococcus aureus*) at concentration 2–4 μg/mL, and merochlorin A was active against *Clostridium difficile* [36].

#### 3.2.4. Terpenoid Phenazines

Bioassay-guided fractionation of fermentation broth of *Streptomyces* sp. CNS-284 and CNY-90 led to isolation of brominated terpenoid phenazine 2-bromo-1-hydroxyphenazine and two novel brominated phenazines (marinocyanin A and B) along with lavanducyanin (nonbrominated phenazine) (Figure 3). Strain CNS-284 was isolated from Palau and strain CNY-90 was isolated from the Solomon Islands. The brominated compounds were active as anti-inflammatory agents and inhibited Tumor Necrosis Factor-α (TNF-α) induced Nuclear factor-kB (NF-kB at IC_50_ 4.1, 24.2, and 16.3 µM, respectively. The compounds also showed activity against Lipopolysaccharide (LPS)-induced NO production at IC_50_ > 48.6, 15.1, and 8.0 μM, respectively. The production Protaglandin E2 (PGE2) was blocked at IC_50_ 7.5, 0.89, and 0.63 μM, respectively. In addition, 2-bromo-1-hydroxyphenazine showed activity in NF-kB-luciferase assay at IC_50_ 73 μM [37].

Marinocyanin A–F were identified from *Streptomyces* CNY-90. Marinocyanin A had potency as antifungal against amphotericin-resistant *Candida albicans* with MIC 0.95 μM. Marinocyanin A and B showed inhibition against cancer cell line HCT-116 at IC_50_ 0.049 μM and 0.029 μM, respectively [38].

#### 3.2.5. Napyradiomycins and Related Compounds

Napyradiomycins are the group of hybrid terpene and polyketide compounds that consist of napthoquinone ring system with halogen substitution. Investigation of broth culture of marine-derived *Streptomyces* SCSIO 10428 from Xieyang Island, China, yielded three new napyradiomycins- related compounds (Figure 3) 4-dehydro-4a-dechloronapyradiomycin A1, 3-dechloro-3-bromo napyradiomycin A1, and 3-chloro-6,8-dihydroxy-8-α-lapachone, isolated along with main products napyradiomycin A1, 18-oxonapyradiomycin A1, napyradiomycin B1, napyradiomycin B3, naphthomevalin, and napyradiomycin SR. Evaluation of their bioactivities showed that they had antibacterial activities with MIC values ranging from 0.5 to 32 μg/mL against *Staphylococcus aureus* ATCC 29213, *Bacillus subtilis* SCSIO BS01, and *Bacillus thuringiensis* SCSIO BT01 but no activity against Gram-negative bacteria. Some displayed activity to human cancer cell line SF-268, MCF-7, NCI-H460, and HepG-2 with IC_50_ values below 20 μM, but some showed activity above 20 μM [39].

Six novel napyradiomycins A–F (Figure 3) along with napyradiomycins B2–B4 were isolated from *Streptomyces* strain CNQ-329 and CNH-070 from sediment in San Diego, California. The strains hade similarity to *Streptomyces aculeolatus*. The napyradiomycins were evaluated against MRSA and HCT-116. Napyradiomycin A was active against MRSA at MIC 16 μg/mL while napyradiomycin B-F were inactive. Napyradiomycin B3 was the most active at MIC 2 μg/mL. Napyradiomycins inhibited HCT-116 at IC_50_ range 4.19–16 μg/mL [40].

Strain *Streptomyces* CNQ-525 produced varied types of napyradiomycin that have been reported. Further investigation of the strain has yielded four new napyradiomycin called CNQ525.510B, CNQ525.538, CNQ525.554, and CNQ525.600 (Figure 3) along with known napyradiomycins such as B1, B3, B4, A80915A, A80915B, A80915C, A80915D, CNQ525.512, and SF2415B3. The compounds were tested against HCT-116 colon carcinoma and showed activity at range less than 1 μM to more than 100 μM [41].

The *Streptomyces* strain CA-271078 associated with ascidian from seashore Baia Ana Chaves, SaoTome, produce a new napyradiomycin MDN-0170 (Figure 3). The compound was inactive against MRSA, *E. coli*, *Aspergillus fumigatus*, and *C. albicans* [42].

### 3.3. Peptides

#### 3.3.1. Piperazimycins

**Figure 4:**
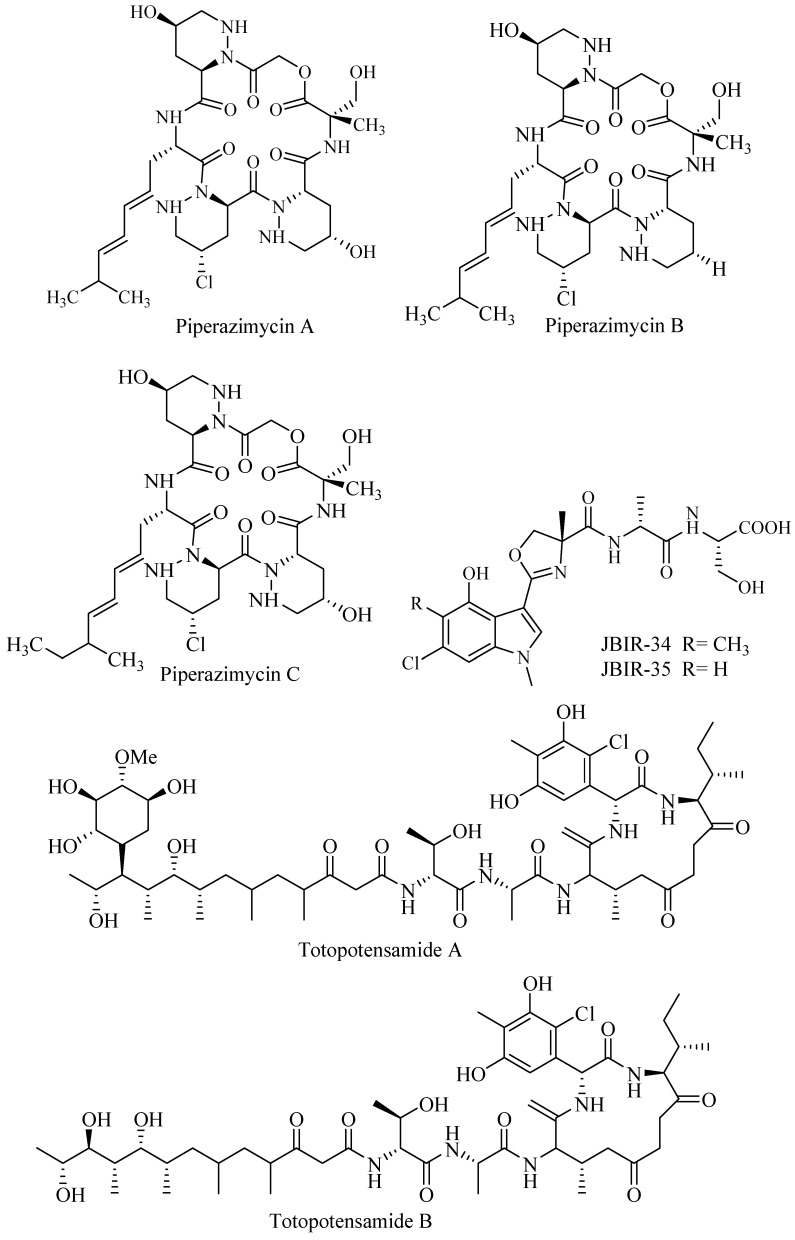
Halogenated peptides.

Cyclic chlorinated hexadepsipeptide piperazimycin A–C (Figure 4) were isolated and purified from an ethyl acetate extract of culture fermentation of *Streptomyces* sp. isolated from Guam. The compounds were assayed for bioactivity against human colon carcinoma and 60 cancer cell lines. Each compound exhibited significant cytotoxicity with an average GI (Growth Inhibition)_50_ 76 ng/mL against HCT-16 (human colon carcinoma). Piperazimycin A was the most potent and 3 times more active against solid tumor compared to other piperazimycins [43].

#### 3.3.2. JBIR

*Streptomyces* Sp080513GE-23 associated with sponge yielded two novel compounds tetrapeptide modified indole named JBIR 34 and JBIR 35 (Figure 4). The compound showed radical scavenging activity at IC_50_ 1.0 and 2.5 mM for JBIR 34 and JBIR 35, respectively [44].

#### 3.3.3. Totopotensamides

*Streptomyces* sp. 1053 U.I. Ia.Ib cultivated from gastropod *Lienardia totopotens* collected near Mactan Island, Cebu, Philippines, produced hybrid peptide-polyketide glycoside totopotensamide A and B (Figure 4). The compounds had interesting features but showed no activity in wide-range bioassay including DRG panel assay for neurological activity [45].

### 3.4. Polyketides

#### 3.4.1. Salinosporamides

**Figure 5:**
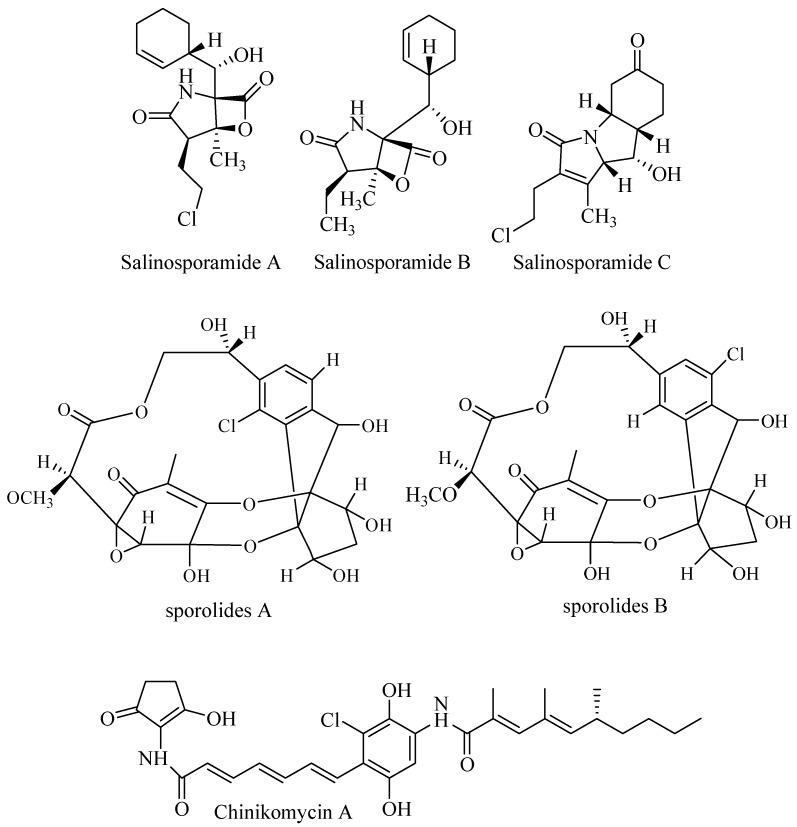

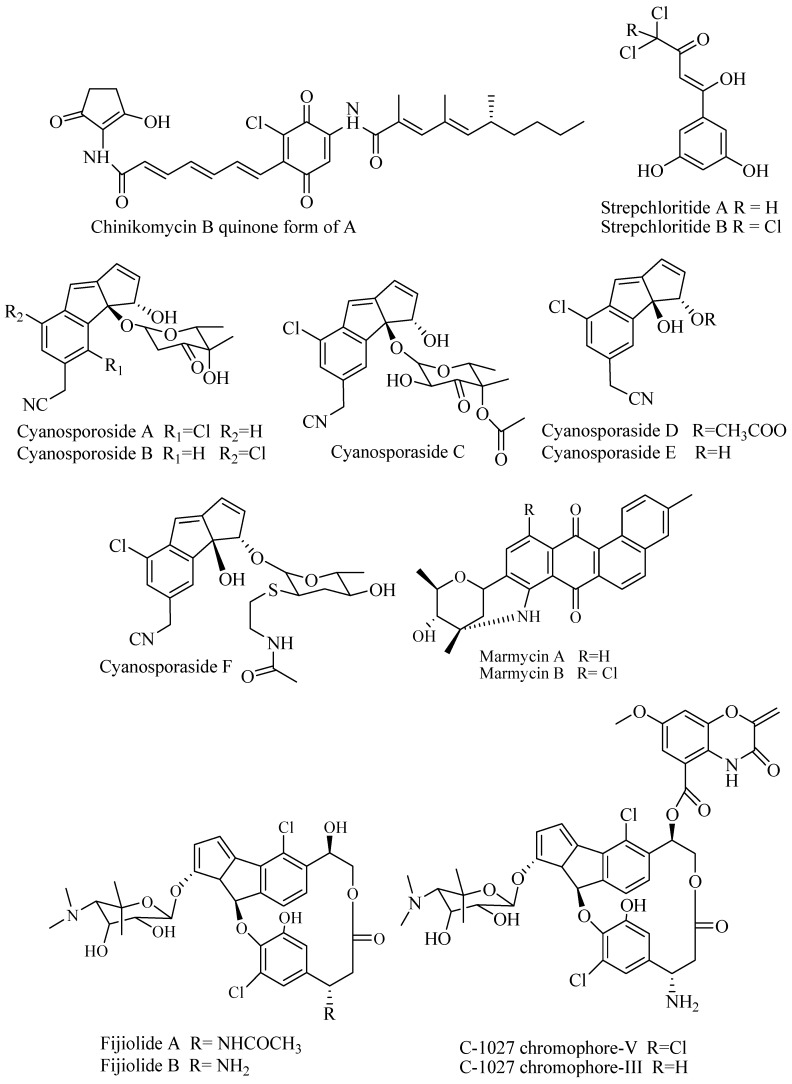
Halogenated polyketide.

Salinosporamide A (Figure 5) was discovered from culture broth of *Salinispora tropica* CNB. The compound has unique and unusual structure consists of fused γ-lactam β-lactone ring structure. Salinosporamide A and B inhibited selectively the proteolytic activity of the 20S subunit of the proteasome. Both compounds also inhibited human colon carcinoma HCT-116 but had no activity against antibiotic-resistant strain *Staphylococcus aureus*, *Enterococcus faecium*, *Candida albicans*, and herpes simplex virus. Salinosporamide A inhibited proteasomal chymotrypsin-like proteolytic at IC_50_ 1.3 nM. Cytotoxicity of salinosporamide A was observed against HCT-116 at IC_50_ 11 ng/mL. The strong potency was examined against NCI-H226 (non-small cell lung cancer), SF-539 (CNS cancer), SK-MEL-28 (melanoma), and MDA-MB-435 (breast cancer) LC_50_ less than 10 nM. Salinosporamide A shares structure similarity to omuralide A but is more potent than omuralide A. This is due to methylation at C-3, chloroethyl group at C-2, and cyclohexene at C-5. Β-lactone moiety is the key for bioactivity. Mechanism of action of salinosporamide A induces apoptosis, suppresses osteoclastogenesis, and inhibits invasion through down-modulation of NF-ĸB regulated gene products [46,47].

#### 3.4.2. Sporolides

Detailed examination of fermentation broth of *Salinispora tropica* CNB-392 (salinosporamide producer) turn out to discover unique polyketide sporolides (Figure 5). These interesting chemical structures were inactive when tested in assay against cancer cell line HCT-116, bacteria MRSA, and Vancomycin Resistance Enterococcus (VRE) [48].

#### 3.4.3. Chinikomycins

Two novel antitumor antibiotic chinikomycins A and B (Figure 5) were isolated from marine *Streptomyces griseoauranticus* M045 along with manumycin A. Chinikomycins were inactive in antibacterial, antiviral, and phytotoxicity assays. Chinikomycin A showed antitumor activity against cell lines MAXF401 NL (mammary), MEXF462 NL (melanoma), and REX (renal cancer) at IC_50_ 2.41, 4.15, and 4.02 μg/mL, respectively. Chinikomycin B was active against MAXF401 at IC_50_ 3.04 μg/mL [49].

#### 3.4.4. Cyanosporasides

Two novel compounds cyanosporaside A and B (Figure 5) were isolated from *Salinispora pacifica* CNS 103 collected from deep-sea sediment in Palau. The cyanosporasides contain 3-keto-pyranohexose sugar and a cyano- and chloro-substituted cyclopenta [a] indene ring. In the bioassay against resistant strain, cyanosporoside A was inactive as antibacterial agent against MRSA, VREF (vancomycin-resistance *Enterococcus faecalis*), and amphotericin-resistant *Candida albicans* (ARCA). The activity against HCT-116 was weak at IC_50_ 30 μg/mL [50]. New derivates (cyanosporasides C–F) were isolated from the marine Actinomycetes *Salinispora pacifica* CNS-143 and *Streptomyces* sp. CNT-179 [51].

#### 3.4.5. Marmycins

Marmycins, an angucycline class of compounds, were isolated from marine Actinomycetes belonging to *Streptomyces* CNH-990. The compounds have no significant activity as antibiotic against MRSA and VREF and antifungal against ARCA. Marmycin A (Figure 5) showed activity against HCT-16 at IC_50_ 60.5 nM, but the chlorinated analog (marmycin B) was less potent at IC_50_ 1.09 μM [52]. This fact is in opposite that halogenation usually responsible for bioactivity and enhance the bioactivity.

#### 3.4.6. Fijiolides

Marine-derived Actinobacteria genus *Nocardiopsis* isolated from sediment near Beqa Island in Beqa Lagoon, Fiji, produce Fijiolide A and B (Figure 5). Fijiolide A enhanced the activity of quinone reductase 1 (QR1), an enzyme that converts quinone to hydroquinone at concentration 28.4 μM. In addition, Fijiolide A reduced TNF-α-induced NF-ĸB activation to 70.3% and IC_50_ 0.57 μM. In contrast, Fijiolide B did not exhibit activities suggesting that substitution on the nitrogen atom affects activity [53]. Arctic marine Actinomycetes identified as *Streptomyces* strain ART 5 was isolated from the arctic region, eastern Siberia during the RV Araon Arctic Expedition (ARA 03B). Profiling chemistry of fermentation broth yielded identification of fijiolide A and B along with articoside, C-1027-chromophore V, and C-1027-chromophore-III. The compounds were tested for bioactivity against *Candida albicans* and antiproliferative activities against human carcinoma cell lines. C-1027-chromophore V and C-1027-chromophore-III showed bioactivity against *C. albicans* at IC_50_ 37.9 μM and 25.6 μM, respectively, but fijiolides showed no activity. The difference in the benzoxazine that counts for the activity. Antiproliferative activities of compounds were ranged from moderate to strong against cancer cell lines HCT-116, A549, SNU638, SK-HEP1, K562, and MDA-MB231 at IC_50_ 0.6–44 μM [54].

#### 3.4.7. Streptochloride

Chlorinated polyketide compound streptochlorides (Figure 5) were isolated from ethyl acetate extract of fermentation broth of *Streptomyces* sp. OUCMDZ-1703 associated with unidentified soft coral. Both compounds have modest antimicrobial activity against *P. aeruginosa*, *E. coli*, and *S. aureus* but no activity against MRSA. Streptochloride A and B demonstrated cytotoxicity against MCF-7 cell line at IC_50_ 9.9 and 20.2 μmol/L, respectively [55].

## 4. Future Direction and Conclusions

Marine Actinobacteria have shown as producer of varied diversity of halometabolites compounds. The compounds range from simple to complicated structures with group under polyketides, peptides, alkaloid, and terpenoid. Some compounds demonstrate intriguing structure special for marine compounds. The compounds exhibited enormous potential for the discovery of new therapeutic leads in the development of drugs to fight the current antibiotic resistance threats, anticancer, and other bioactivities. Marine Actinobacteria produce more chlorometabolites than bromometabolites in contrast with sponges and red algae which are rich in bromometabolites.

To date, there are several bioprospecting programs with target marine biodiversity for novel bioactive metabolites including halometabolites. FADH_2_-dependent halogenase is the biggest group of halogenating enzymes, thus can be used as target in the bioprospecting of halometabolites from marine Actinobacteria. Genome mining by employing gene that encodes FADH_2_-dependent halogenase as an indicator has enabled to screen 555 genetic potentials of actinomycetes for halogenated natural products [56]. Gao and Huang employed the same approach to screen 228 Actinomycetes to find distribution of the gene and secondary metabolites [57]. Screening mangrove-derived Actinomycetes using FADH_2_-dependent halogenase resulted in 26 halogenase-positive strain among 163 isolates [58]. PCR-based marker gene screening was employed to detect FADH_2_-dependent halogenase gene of Arctic marine Actinobacteria. The study concluded that Arctic marine Actinobacteria are potential in halometabolites production [59]. Three novel halogenase gene clusters were identified in microbial metagenome of marine sponge indicated that the microbial consortia of sponges including marine Actinobacteria are a valuable resource for novel halogenation [60]. There is a correlation between the distribution of FADH_2_-dependent halogenase gene in filamentous actinomycetes and the potential for producing halometabolites.

Comparative genome studies showed that Actinobacteria are rich in secondary metabolites genes that never been explored, so the chance to discover new bioactive halometabolites is still wide open. Further research into mechanisms of biological halogenation will provide insight and a greater understanding of biosynthesis of halometabolites. Furthermore, understanding the genes encoding halogenase enzymes may be used to generate recombinant organisms to produce derivative new natural product. Advance technology in exploration and collection, compound isolation, purification, structure elucidation, bioassay, and high-throughput screening will ensure and enable to identify potential halometabolites from marine Actinobacteria for benefit to humankind.
